# Supplementary material for: Evaluation of Wheat Germplasm for Resistance to Leaf Rust (Puccinia triticina) and Identification of the Sources of Lr Resistance Genes Using Molecular Markers
Source: Plants (Basel). 2021 Jul 20;10(7):1484. doi: 10.3390/plants10071484 (PMC8309318; doi:10.3390/plants10071484)
Supplement: Supplementary file 1 [file plants-10-01484-s001.zip › plants-1280731-supplementary.pdf]

Table S1. Seedlings reaction of Thatcher *Lr* genes isogenic lines to eight *Prt* pathotypes, Kazakhstan.

Thatcher Near Isogenic Line	Seedlings Reaction to Leaf Rust Pathotypes:							
	TKT/Q	KHT/B	TGT/G	TLT/R	SBR/H	THT/B	QBQ/G	SBP/C
Tc*Lr1*	4	2+	4	3+	3+	3+	3+	3+
Tc*Lr2a*	3+	3+	4	4	3+	3+	4	3+
Tc*Lr2c*	3	3+	3+	3+	3+	3+	;1−	3+
Tc*Lr3a*	3+	3	3+	3+	2+	3+	2+	2+
Tc*Lr3bg*	4	3	3+	3+	2+	3+	2+	2+
Tc*Lr3ka*	3	3+	3+	3+	3+	3+	3+	3+
Tc*Lr9*	0	0	0	3	0;	0	0;	0
Tc*Lr10*	;1	2+	3+	3+	2+	;1	0;	;1−
Tc*Lr11*	3+	3+	3	3	3+	3+	3+	2+
Tc*Lr14b*	3	2	3+	3+	2+	3+	3+	3+
Tc*Lr16*	4	3	3+	;1	;1−	3+	;1−	;1−
Tc*Lr17*	3+	3	3+	3+	2+	3	2+	3+
Tc*Lr18*	3	2	3+	3+	2	3+	2+	;1
Tc*Lr19*	3+	0	2	3	2	;1−	0	0
Tc*Lr20*	3+	2	3+	3+	3+	;1−	3+	2+
Tc*Lr21*	3+	2+	4	3+	2+	3+	0	0
Tc*Lr23*	3+	;1	2	;1	2+	;1−	0	2+
Tc*Lr24*	3+	;1−	0;	;	0	2+	0;	2+
Tc*Lr25*	0	0;	0	0	0;	0;	0;	0;
Tc*Lr26*	3+	3	2	;1−	2+	3+	2+	2+
Tc*Lr29*	2	2	;1−	3+	3+	2+	2+	3+
Tc*Lr30*	3+	3	3+	3+	3+	3+	2+	3+

Table S2. Markers and primers used to identify the presence of *Lr* genes in wheat germplasm.

Gene	Localization	Primer Name	Primer Sequence (5′–3′)	Annealing Temp. (°C)	Fragment Size (bp)	Reference
Lr1	5DL	pTAG 621-3<br>pTAG 621-5	5′-CCT TGC CAG CCC AAA AG-3′<br>5′-GGG TCA CGT ACT ACT ATA-3′	55	560 bp	[47]
Lr9	6B	J13/1<br>J13/2	5′-TCC TTT TAT TCC GCA CGC CGG-3′<br>5′-CCA CAC TAC CCC AAA GAG ACG-3′	62	1100 bp	[48]
Lr10	1AS	F1.2245<br>Lr10-6/r2	5′-GTG TAA TGC ATG CAG GTT CC-3′<br>5′-AGG TGT GAG TGA GTT ATG TT-3′	57	310 bp	[49]
Lr19	7A	PSY1-EF2<br>PSY1-ER4	5'-CTA CGT TGC GGG CAC CGT T-3'<br>5'-AGA GAA AAC CAT TGC ATC TGT A-3'	63	191 bp	[50]
Lr26	1BL	SCM9	5'-TGACAACCCCCTTTCCCTCGT-3′<br>5'-TCATCGACGCTAAGGAGGACCC-3′	60	207 bp	[51]
Lr28	4AL	Wmc313	5′-GCA GTC TAA TTA TCT GCT GGC G-3′<br>5′-GGG TCC TTG TCT ACT CAT GTC C-3′	51	320 bp	[52]
Lr34	7D	csLV34-F<br>csLV34-R	5′-GTT GGT TAA GAC TGG TGA TGG-3′<br>5′-TGC TTG CTA TTG CTG AAT AGT-3′	55	150 bp<br>229 bp	[53]
Lr37	*2AS*	Ventriup<br>Ln	5'-AGGGGCTACTGACCAAGGCT-3'<br>5'-<br>TGCAGCTACAGCAGTATGTACACAAAA-<br>3'	65	262 bp	[54]
Lr68	7BL	csGS-F1<br>csGS-R1	5'-AAG ATT GTT CAC AGA TCC ATG TCA-<br>3'<br>5'-GAG TAT TCC GGC TCA AAA AGG-3'	60	385 bp	[55]
